# Spontaneous giant rectus sheath hematoma in patients with COVID-19: two case reports and literature review

**DOI:** 10.1186/s12245-021-00366-5

**Published:** 2021-07-23

**Authors:** Behzad Nematihonar, Shohra Qaderi, Jaffer Shah, Javad zebarjadi Bagherpour

**Affiliations:** 1 Department of General Surgery, School of Medicine, Imam Hossein Hospital, Shahid Beheshti University of Medical Sciences, Madani Street, Tehran, 161776341 Iran; 2 Student Research Committee, School of Medicine, Shahid Beheshti University of Medical Sciences, Tehran, Iran; 3 Medical Research Center, Kateb University, Kabul, Afghanistan; 4 Drexel University College of Medicine, Philadelphia, PA USA

**Keywords:** COVID-19, Vascular disease, Sudden adnominal pain, Muscle hematoma, Case report, Rectus sheath hematoma

## Abstract

**Introduction:**

Coronavirus disease 2019, COVID-19, as a global public health emergency, has come with a broad spectrum of clinical manifestations and complications. In this study, we present a unique complication of this disease.

**Presentation of cases:**

(A) A 65-year-old woman with a known case of COVID-19; on the second day of admission, the patient presented sudden tachycardia and hypogastric pain; on abdomen physical examination, a huge lower abdominal tender mass was noticed. (B) A 50-year-old woman with COVID-19, 4 days after admission, started complaining of tachycardia, pain, and mass in the lower abdomen. On abdomen physical examination, a huge lower abdominal tender mass was noticed. Both of the patients underwent an abdomen CT scan which confirmed a huge rectus sheath hematoma (RSH). Both of the patients underwent angioembolization of the inferior epigastric artery. The patient recovered completely and no evidence of further expansion was seen after 2 weeks of follow-up.

**Discussion:**

Hemorrhagic issues in COVID-19 patients remain poorly understood. Physicians should discuss risks of RSH in patients where continuous anticoagulation therapy will be reinstated. With increased clinician awareness of the need for RSH screening in COVID-19 patients with acute abdominal pain, the interprofessional team of healthcare providers can maximize patient safety and reduce hospitalization time, especially in high-risk patients at risk for unnecessary surgery.

**Conclusions:**

These two reports and literature review demonstrate the need of active surveillance for possible hemorrhagic complications in patients with COVID-19 infection.

## Background

The superior epigastric artery lies deep into the rectus abdominis muscles and continues to perforate and supply the rectus sheath. Sudden and violent contraction of the rectus abdominis muscles may cause the epigastric arteries to tear. Rectus sheath hematoma (RSH) is a rare complication seen in patients with acute abdominal pain with a mass present in the abdominal wall which may result from several cases, mostly in the use of anticoagulant and/or anti-aggregant medications for various reasons [3, 4, 19]. Coronavirus disease 2019, COVID-19, as a global public health emergency, has come with a broad spectrum of clinical manifestations which ranges from fever, fatigue, myalgia, cough, and dyspnea to pneumonia, and even acute respiratory distress syndrome. Extrapulmonary involvements including gastrointestinal, cardiovascular, and renal to unusual presentations such as encephalitis, acute limb ischemia, and tremors and gait disturbance have been reported as well [13, 17]. Endothelial dysfunction, thrombosis, and deregulated inflammation are reported as associated vascular diseases in patients with SARS-Cov-2 [11, 13]. Due to this reason, anticoagulant therapy with low molecular weight heparin (LMWH) has become part of medical therapy in hospitalized patients with COVID-19. This approach may increase the risk of spontaneous bleeding, epically in elderly patients with comorbidities, which highlights the importance of active surveillance of spontaneous bleeding, which requires the active involvement of medical staff in the emergency setting [1, 16]. Here, we present giant RSH in two confirmed cases of COVID-19 patients, who were under a prophylactic dose of anticoagulation.

## Case presentation

### Case 1

**Fig. 1:**
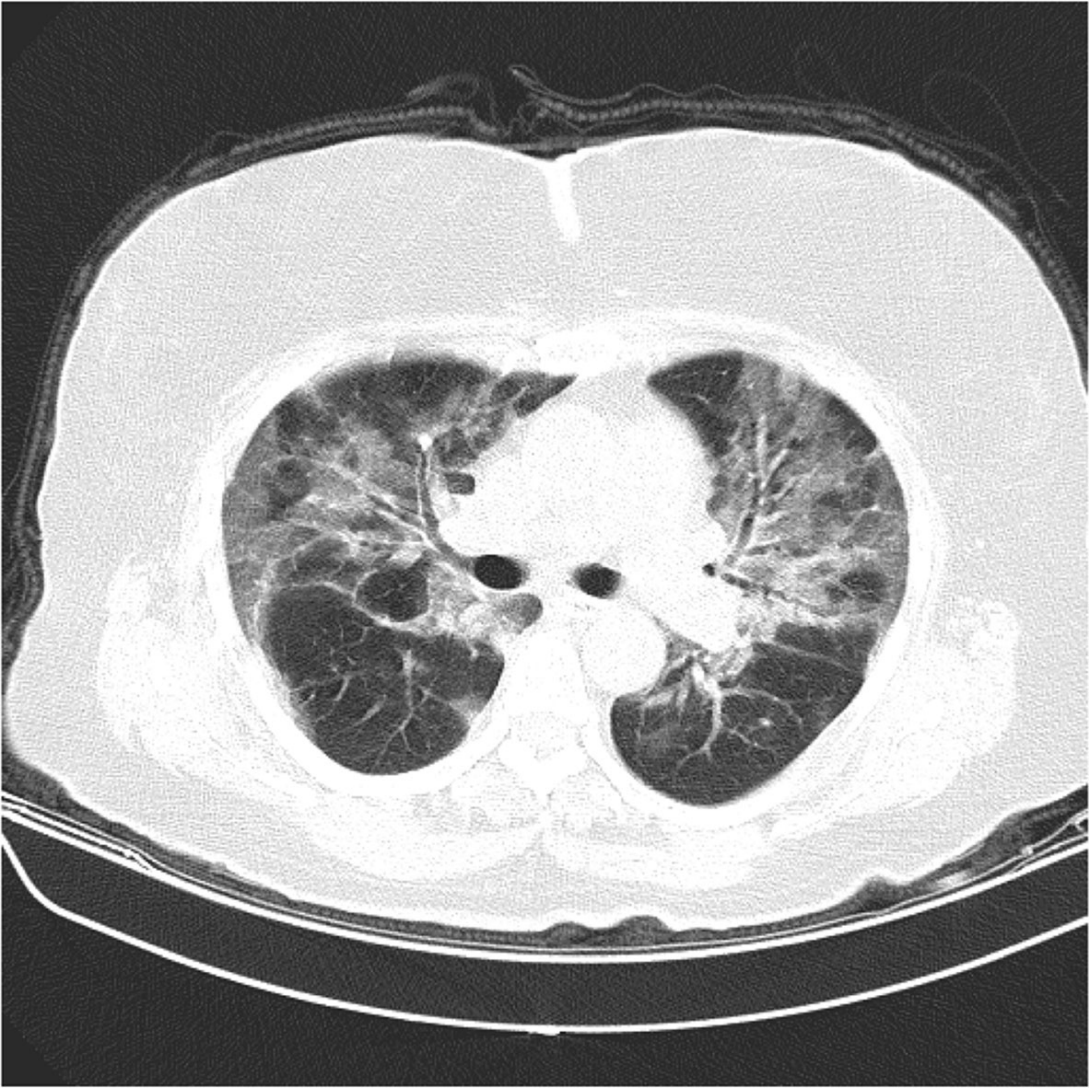
Chest CT scan shows signs of bilateral COVID-19 pneumonia

**Fig. 2:**
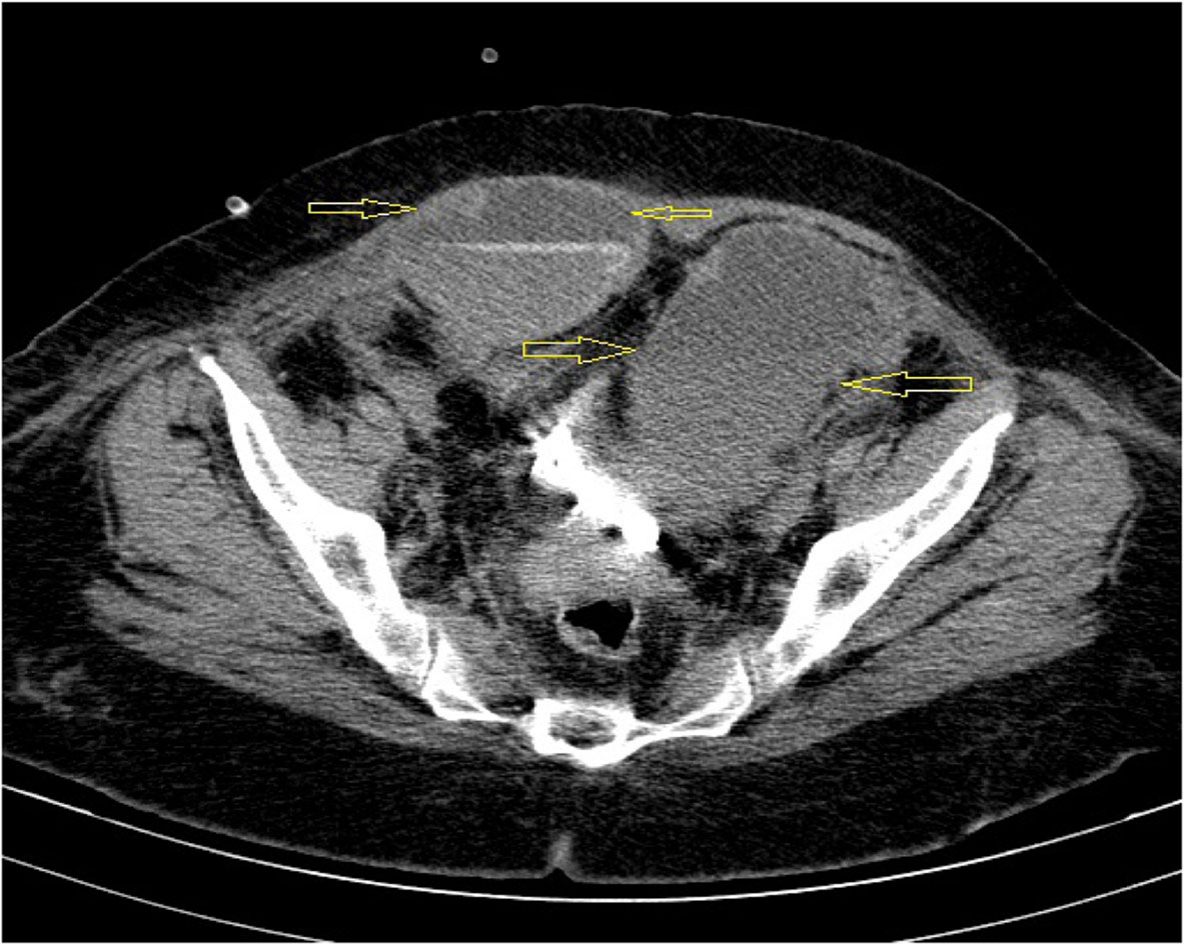
The yellow arrows in the image confirm a huge rectus sheath hematoma

A 65-year-old woman with a history of diabetes mellitus type 2 on metformin therapy presented to the respiratory emergency department after experiencing a sudden onset of dyspnea on mild exertion. Concomitantly, she was complaining of severe nonproductive cough, fatigue, and weakness for about 4 days of duration. At admission, the patient was afebrile and eupneic in oxygen therapy (pulse oxygen saturation = 95% with simple mask flow 2–4 L/min). Blood investigation revealed a total leucocyte count of 15,000/μL (normal 4000 to 11,000/μL), hemoglobin (HB) 12 g/dL (normal 13 to 18 g/dL), platelet 444 × 10^3^ (normal 140 × 10^3^ to 400 × 10^3^), partial thromboplastin time (PTT) 32 (normal 23 to 35 s), international normalized ratio (INR) 1.4 (normal 0.8 to 1.1), erythrocyte sedimentation rate (ESR) 110 mm/1 h (normal up to 14 mm/h), and C-reactive protein (CRP) 100 (normal < 10 mg/L). The result of the RT-PCR test was positive for SARS-Cov-2, and the chest computed tomography (CT) scan revealed bilateral ground-glass opacities (GGO) (Fig. 1). The patient was admitted to the COVID-19 ward, and based on the local protocol, remdesivir, azithromycin, dextromethorphan (due to violent cough), and prophylactic dose of heparin (5000 every 8 h) had begun. The second day after admission, the patient presented sudden tachycardia and hypogastric pain; on abdomen physical examination, a huge lower abdominal tender mass was noticed (blood pressure 100/80 mmHg, pulse rate 100/min, and respiratory rate 19/min) and there was a 2-g/dL decrease in HB from 12 to 10 g/dL. The patient underwent an abdomen CT scan which confirmed a huge rectus sheath hematoma with no extension to other parts of the body (Fig. 2). On the same day, the patient underwent angioembolization of the inferior epigastric artery. Overall, the patient received isotonic fluids and the trend of blood investigation is shown in Table 1. After 6 days of embolization, she recovered completely and no evidence of further expansion was seen. She was discharged, and after 2 weeks of follow-up, she did not develop any complication.

**Table 1:** The result of blood tests during hospitalization

Tests	First day	Second day	Sixth day	Reference range
**WBC**	15,000	14,000	8000	4000–11,000/μL
**HB**	12	10	11	13–18 g/dL
**PLT**	444	351	310	140 × 10^3^–400 × 10^3^
**PTT**	32	25	31	23 –35 s
**INR**	1.4	1.2	1.4	0.8–1.1
**ESR**	110	114	12	Up to 14 mm/h
**CRP**	100	75	18	< 10 mg/L

### Case 2

**Fig. 3:**
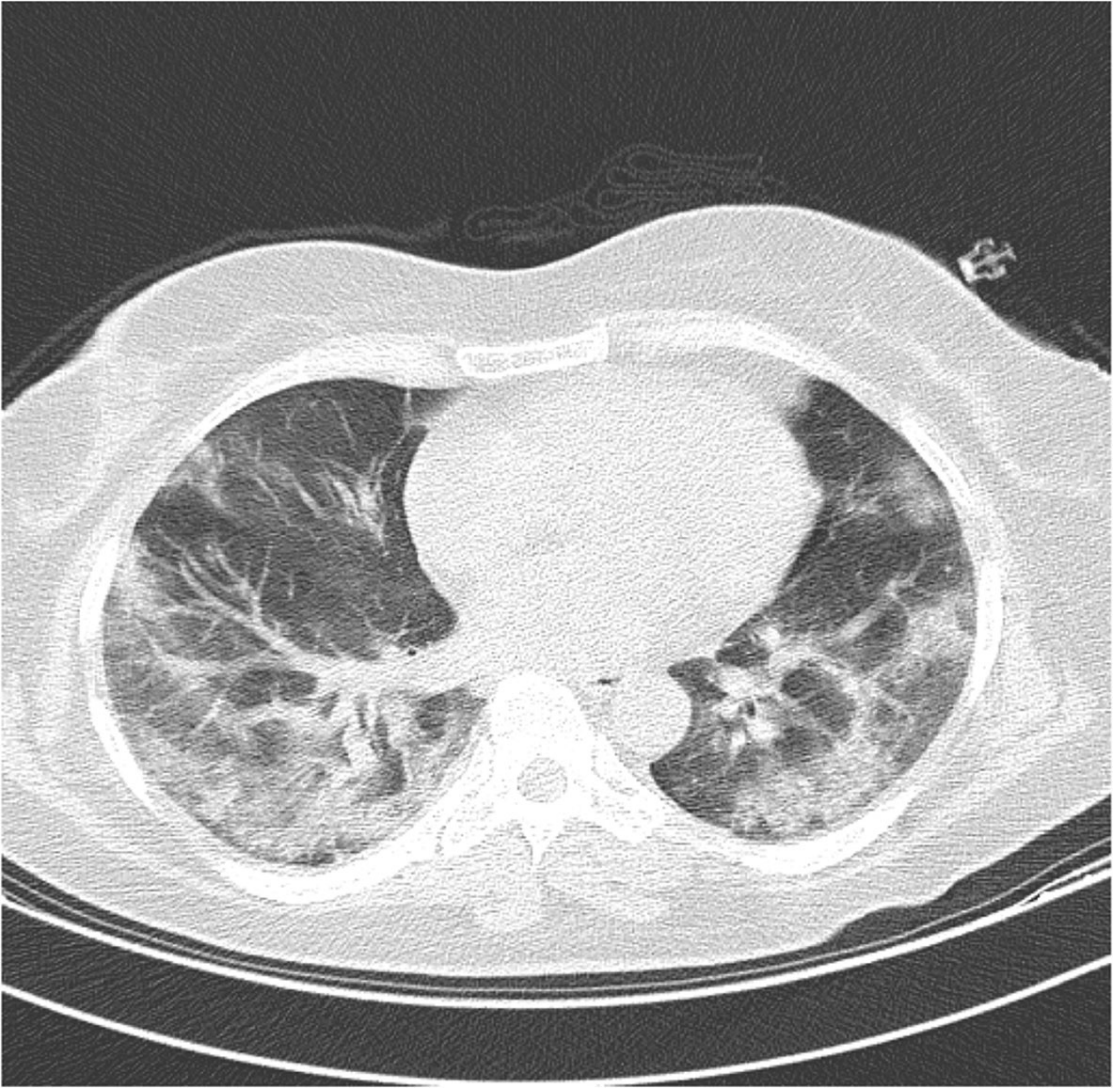
The chest CT scan showing typical GGO features in COVID-19 pneumonia

**Fig. 4:**
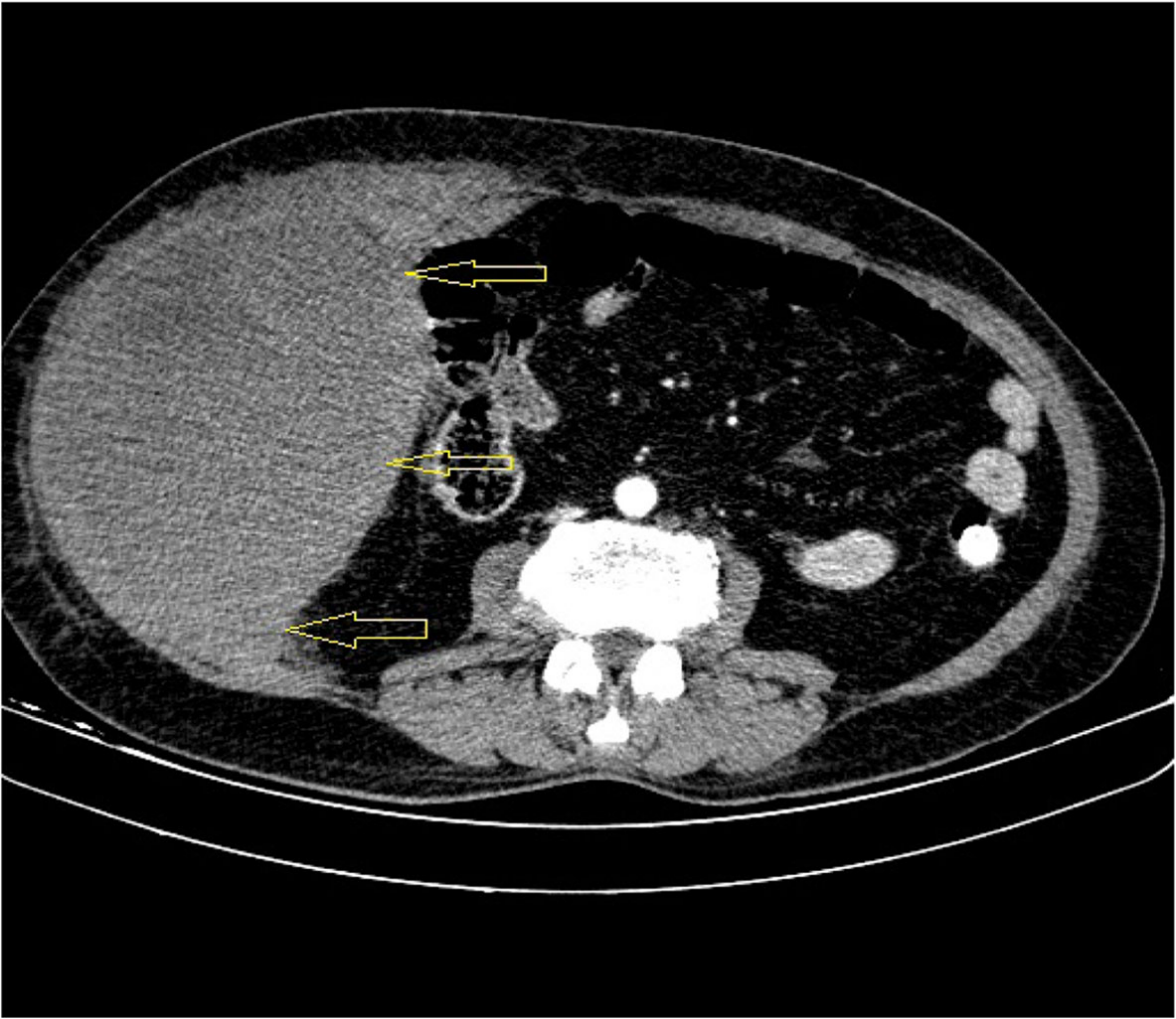
The yellow arrows in the image revealed a huge rectus sheath hematoma

A 50-year-old woman presented to the respiratory emergency department with a chief complaint of shortness of breath on exertion. She reported severe nonproductive cough, fever, and fatigue for about 6 days ago. She has no history of trauma and the patient was otherwise in good health. On physical examination, a decreased breath sounds in the lower lobes of the lungs was heard, bilaterally. At that time, she was febrile (temperature 39 °C) and eupneic in oxygen therapy (pulse oxygen saturation = 96% with nasal oxygen 2–4 L/min). Laboratory blood investigation revealed a total leucocyte count of 14,000/μL (normal 4000 to 11,000/μL), hemoglobin (HB) 12 g/dL (normal 13 to 18 g/dL), platelet 368 × 10^3^ (normal 140 × 10^3^ to 400 × 10^3^), partial thromboplastin time (PTT) 29 (normal 23 to 35 s), international normalized ratio (INR) 1.2 (normal 0.8 to 1.1), erythrocyte sedimentation rate (ESR) 154 mm/1 h (normal up to 14 mm/h), and C-reactive protein (CRP) 75 (normal < 10 mg/L). The result of the RT-PCR test was positive for SARS-Cov-2, and the chest computed tomography (CT) scan revealed bilateral ground-glass opacities (GGO) (Fig. 3). The patient was admitted to the COVID-19 ward, and based on the local protocol, remdesivir, azithromycin, dextromethorphan (due to violent cough), and prophylactic dose of subcutaneous enoxaparin (40 mg every 24 h) had begun. Suddenly, 4 days after admission, the patient started complaining of tachycardia, pain, and mass in the lower abdomen. On abdomen physical examination, a huge lower abdominal tender mass was noticed (blood pressure 120/90 mmHg, pulse rate 110/min, and respiratory rate 19/min). Laboratory tests showed a fall in Hb from 12 to 8 g/dL. Coagulation factors such as PTT, INR, and platelet were in the normal range at that time (Table 2). Overall, the patient was transfused 4 units of packed blood cells, which raised her Hb to 11 g/dL. The patient underwent an abdomen CT scan which confirmed a huge rectus sheath hematoma with no extension to other parts of the body (Fig. 4). The next day, she underwent angioembolization of the inferior epigastric artery. The trend of blood investigation is shown in Table 2. After 6 days of embolization, she recovered completely and no evidence of further expansion was seen. She was discharged, and after 3 weeks of follow-up, she did not develop any complication.

**Table 2:** The result of blood tests during hospitalization

Tests	First day	Fourth day	Seventh day	Reference range
**WBC**	14,000	9000	6000	4000–11,000/μL
**HB**	12	8	11.5	13–18 g/dL
**PLT**	368	271	310	140 × 10^3^–400 × 10^3^
**PTT**	29	20	31	23–35 s
**INR**	1.2	1	1.4	0.8–1.1
**ESR**	154	102	25	Up to 14 mm/h
**CRP**	75	85	12	< 10 mg/L

## Discussion

Previous studies have documented that RSH frequency is greatest in women over 60, likely due to greater rectus sheath muscle mass in men which provides a degree of cushioning to the epigastric arteries from injury [3, 6]. Common risk factors and causes for RSH include anticoagulant therapy, violent coughing, pregnancy, and trauma [5, 6]. A study at the Mayo Clinic found 70% of patients treated for RSH were undergoing anticoagulant therapy [3]. Patients with RSH typically present with acute abdominal pain, sometimes severe and exacerbated by movements involving abdominal wall contraction. Upon physical examination, RSH patients will exhibit tenderness along the rectus sheath. A palpable abdominal mass has been reported in 63–92% of cases, along with abdominal guarding in 49% of RSH cases [8]. The literature demonstrates that other conditions such as ectopic pregnancy, diverticulitis, incarcerated hernia, and abdominal aneurysm can mimic the presentation of RSH [7, 10]. In patients hemodynamically unstable from the RSH, hypotension and tachycardia may also be observed. Various physical examination procedures have previously been described to distinguish intra-abdominal from abdominal wall origin of the tenderness [12]. Carnett’s sign is one such test whereby increased or unchanged tenderness upon tensing the abdominal muscles suggests likely abdominal wall pathology. A negative Carnett’s test is when tenderness decreases upon tensing the abdominal muscles, likely indicating intra-abdominal pathology [18].

As with all cases of hemorrhage, monitoring hematocrit levels is essential to determine if transfusion is necessary. Hematocrit levels in RSH patients vary depending on the severity, with normal values in small RSH to large reductions in hematocrit levels reported in cases of large RSH [9]. Most cases of RSH are self-limiting and may be managed successfully without the need for invasive surgery [3]. Recovery from RSH can last between 2 and 3 months and most patients recover with no complications [3]. Mortality rates are estimated to be 4%, but rates as high as 25% have been observed in patients on anticoagulation therapy. Angioembolization is the preferred intervention when persistent bleeding is observed [15]. Patients receiving oral anticoagulation must have coagulation factors monitored, and treatment can involve either cessation of the anticoagulation therapy or anticoagulation reversal.

Ultrasonography (US) sensitivity is limiting for RSH diagnosis [14]. Studies have demonstrated US is non-specific and poses a challenge to distinguish RSH from abdominal wall tumors. Abdominal CT, with 100% sensitivity, is preferred over US for the diagnosis of RSH, although US may be preferred in certain patient populations with kidney disease [8]. CT imaging typically reveals a spindle-shaped mass posterior to the abdominal muscles. CT findings are used to classify RSH into three types that are mild, moderate, and severe. Type 1 is mild and does not require hospitalization, while type 3, typically associated with anticoagulation, is severe and requires hospitalization with transfusion and hemodynamic stabilization [2].

## Conclusions

This case was a rare example of a RSH in a confirmed COVID-19 patient. Although the hypercoagulable state associated with SARS-Cov-2 infection has received much recent attention, hemorrhagic issues in COVID-19 patients remain poorly understood. Physicians should discuss risks of recurrent RSH in patients where continuous anticoagulation therapy will be reinstated. With increased clinician awareness of the need for RSH screening in COVID-19 patients with acute abdominal pain, the interprofessional team of healthcare providers can maximize patient safety and reduce hospitalization time, especially in high-risk patients at risk for unnecessary surgery.
